# Nuclear factor Y-A3b binds to the *SINGLE FLOWER TRUSS* promoter and regulates flowering time in tomato

**DOI:** 10.1093/hr/uhae088

**Published:** 2024-04-02

**Authors:** Dedi Zhang, Kangna Ji, Jiafa Wang, Xinyu Liu, Zheng Zhou, Rong Huang, Guo Ai, Yan Li, Xin Wang, Taotao Wang, Yongen Lu, Zonglie Hong, Zhibiao Ye, Junhong Zhang

**Affiliations:** National Key Laboratory for Germplasm Innovation & Utilization of Horticultural Crops, Huazhong Agricultural University, Wuhan 430070, China; National Key Laboratory for Germplasm Innovation & Utilization of Horticultural Crops, Huazhong Agricultural University, Wuhan 430070, China; College of Horticulture, Northwest A&F University, Yangling 712100, Shaanxi, China; National Key Laboratory for Germplasm Innovation & Utilization of Horticultural Crops, Huazhong Agricultural University, Wuhan 430070, China; National Key Laboratory for Germplasm Innovation & Utilization of Horticultural Crops, Huazhong Agricultural University, Wuhan 430070, China; National Key Laboratory for Germplasm Innovation & Utilization of Horticultural Crops, Huazhong Agricultural University, Wuhan 430070, China; National Key Laboratory for Germplasm Innovation & Utilization of Horticultural Crops, Huazhong Agricultural University, Wuhan 430070, China; Zhumadian Academy of Agricultural Sciences, Zhumadian 463000, China; National Key Laboratory for Germplasm Innovation & Utilization of Horticultural Crops, Huazhong Agricultural University, Wuhan 430070, China; National Key Laboratory for Germplasm Innovation & Utilization of Horticultural Crops, Huazhong Agricultural University, Wuhan 430070, China; National Key Laboratory for Germplasm Innovation & Utilization of Horticultural Crops, Huazhong Agricultural University, Wuhan 430070, China; Department of Plant Sciences, University of Idaho, Moscow, ID 83844, USA; National Key Laboratory for Germplasm Innovation & Utilization of Horticultural Crops, Huazhong Agricultural University, Wuhan 430070, China; National Key Laboratory for Germplasm Innovation & Utilization of Horticultural Crops, Huazhong Agricultural University, Wuhan 430070, China

## Abstract

The control of flowering time is essential for reproductive success and has a major effect on seed and fruit yield and other important agricultural traits in crops. Nuclear factors Y (NF-Ys) are transcription factors that form heterotrimeric protein complexes to regulate gene expression required for diverse biological processes, including flowering time control in plants. However, to our knowledge, there has been no report on mutants of individual NF-YA subunits that promote early flowering phenotype in plants. In this study, we identified *SlNF-YA3b*, encoding a member of the NF-Y transcription factor family, as a key gene regulating flowering time in tomato. Knockout of *NF-YA3b* resulted in an early flowering phenotype in tomato, whereas overexpression of *NF-YA3b* delayed flowering in transgenic tomato plants. NF-YA3b was demonstrated to form heterotrimeric protein complexes with multiple NF-YB/NF-YC heterodimers in yeast three-hybrid assays. Biochemical evidence indicated that NF-YA3b directly binds to the CCAAT *cis*-elements of the *SINGLE FLOWER TRUSS* (*SFT*) promoter to suppress its gene expression. These findings uncovered a critical role of *NF-YA3b* in regulating flowering time in tomato and could be applied to the management of flowering time in crops.

## Introduction

Flowering is an important transition in flowering plants from vegetative to reproductive growth. In agricultural production, flowering is not only an important stage for the transfer of genetic material from parental plants to their offspring but also a prerequisite to producing fruits and seeds [[4]]. The timing of floral transition plays an essential role in the control of plant fertility, plant yield quality, and other important agricultural traits in crops.

In *Arabidopsis*, the FLOWERING LOCUS T (FT) protein is widely believed to be the key component of the elusive flowering hormone florigen, which plays a pivotal role in flowering time control. FT serves as the long-distance signal that is expressed in the phloem cells of leaf veins and transported from leaves to the shoot apex to induce the initiation of floral primordia in *Arabidopsis* [10, 19]. *Heading date 3a* (*Hd3a*), the rice ortholog of *Arabidopsis FT*, plays a similar role in the induction of flowering in rice [48]. In the shoot apex, FT generates a protein complex with a 14-3-3 protein and FLOWERING LOCUS D (FD) to promote flowering [49, 55]. Tomato *SINGLE FLOWER TRUSS* (*SFT*) is a homolog of *Arabidopsis FT* and its overexpression in transgenic tomato plants leads to early flowering, as is the case for *FT* overexpression in *Arabidopsis* [50]. In addition, *SFT* has an effect on the development of flower and inflorescence morphology in tomato. The *sft* mutant plants not only have a delayed flowering phenotype but also develop a single inflorescence with flowers having large sepals [31].

NF-Y transcription factors are sometimes referred to as CCAAT-binding factors (CBFs) or hemo-activator proteins (HAPs). They are a highly prevalent and evolutionary conserved class of transcription factors that are found in yeast, animals, and plants [11]. According to the presence of different structural features, NF-Y subunits can be classified into three major groups, NF-YA (HAP2/CBF-B), NF-YB (HAP3/CBF-A), and NF-YC (HAP5/CBF-C) [24]. The tomato genome has the most *NF-Y* genes, containing 10 *NF-YA*s, 29 *NF-YB*s, and 20 *NF-YC*s [28]. In plants, NF-YB and NF-YC subunits form dimers through the interaction between their histone folding domains (HFDs) [15, 42], subsequently recruiting NF-YA to form an NF-Y heterotrimeric protein complex [20]. NF-YA can recognize and bind to specific CCAAT-box *cis*-elements of promoters and enhancers from target genes [6]. Several studies have demonstrated that NF-Ys are essential for the development of symbiotic root nodules, flavonoid biosynthesis, photomorphogenesis, photosynthesis, abscisic acid (ABA)-regulated seed germination, response to stress, and reproductive development [7, 22, 23, 25, 40, 41, 43].

The NF-Y transcription factor family plays a crucial role in flowering regulation [3, 16, 24, 41, 54]. It has been demonstrated that overexpression of a series of individual NF-Y subunits, including *NF-YA1*/*4*/*8*, *NF-YB1*/*2*/*3*, and *NF-YC1*/*2*/*3*/*4*/*9*, can change flowering times in transgenic plants [7, 16–18, 22, 23, 47, 62]. The DNA binding domain of CONSTANS (CO) is homologous to that of NF-YA, and various NF-YB and NF-YC subunits can interact with CO to form NF-Y/CO heterotrimeric complexes that mediate CO-regulated flowering processes [1, 14, 24, 58]. Further studies suggest that the NF-YB/NF-YC dimer interacts with CO, Heading Date1 (HD1), and other proteins that contain the structural domain of TIMING of CAB EXPRESSION1 (CCT), and this distinctive structure of CCT allows its specific targeting to the conserved CCACA motif from the promoters of its target genes to regulate flowering [8, 44]. Furthermore, the C-terminal CCT structural domain of CO forms a complex with NF-YB/NF-YC that recognizes multiple *cis*-elements in the *FT* promoter, and the N-terminal tandem B-box structural domain exhibits a head-to-tail oligomeric conformation to form a homopolymer and mediates *FT* activation, and these multivalent bindings give the CO-NF-Y complex high affinity and specificity [35, 60]. Overexpression of *AtNF-YA1* and *AtNF-YA4* may compete with CO for binding to the NF-YB/NF-YC dimer, resulting in reduced transcript levels of *FT* and causing delayed flowering [26, 33, 37]. Moreover, overexpression of *AtNF-YC1* and *AtNF-YC2* results in elevated transcript levels of *FT* and accelerated flowering process, and, vice versa, mutations in the *AtHAP3b* (an *NF-YB* gene) gene lead to down-regulated transcript levels of *FT* and delayed flowering time [16]. In rice, *OsNF-YB11* (*DTH8*/*Ghd8*/*LHD1*) suppresses the expression of flowering-associated genes and delays photoperiod-induced flowering [12, 53]. Overexpression of *HvNF-YB1* in barley is found to promote early flowering. In wheat, NF-Y interacts with Vernalization Gene 2 (VRN2) and Constans 2 (CO2) *in vivo* and *in vitro*, playing an important role in integrating vernalization and photoperiodic signals to regulate the flowering time [29]. In addition, NF-Y transcription factors participate in the gibberellic acid (GA) signaling pathway-mediated flowering time control and the microRNA-mediated age-dependent flowering time regulation [17, 52, 61, 62].

Previous studies on the effect of NF-Y on flowering time have mainly been focused on model plants such as *Arabidopsis* and rice. To our knowledge, there have been few studies on the effect of *NF-YA* transcription factors on flowering time control in tomato. Here, we report that *NF-YA3b* negatively regulated flowering time by binding to the CCAAT *cis*-element of the *SFT* promoter. As compared to wild-type (WT) tomato plants, *NF-YA3b* knockout lines had significantly earlier flowering time, and the *SFT* transcript level was also significantly up-regulated. We demonstrated that NF-YA3b was recruited by multiple NF-YB/NF-YC heterodimers to generate heterotrimeric complexes in yeast three-hybrid (Y3H) assays. The direct binding of NF-YA3b to the CCAAT *cis*-element of the *SFT* promoter was verified in the yeast one-hybrid (Y1H) system, the dual luciferase reporter system, and the electrophoretic mobility shift assay (EMSA). These findings demonstrate the critical role of *NF-YA3b* in regulating flowering time in tomato and provide an opportunity for using the *NF-YA3b* gene as a target of genetic manipulation for flowering time management in crops using the CRISPR/Cas9 system.

## Results

### Characterization of *SlNF-YA3b*

**Figure 1:**
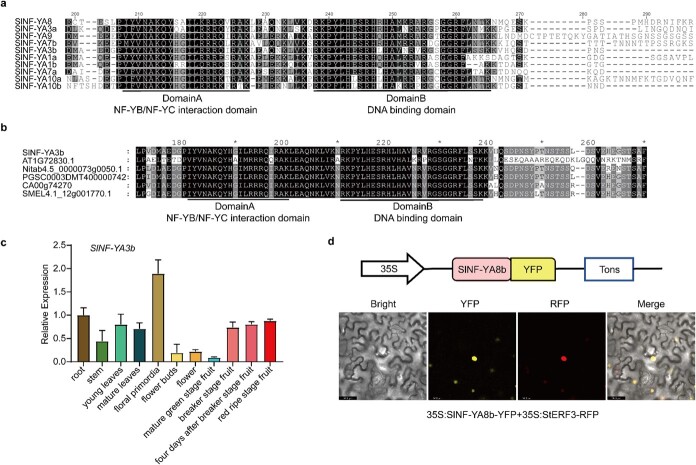
Characterization of SlNF-YA3b. **a**, **b** Protein sequence alignments of 10 tomato NF-YA members (**a**) and representatives of NF-YA counterparts from other plant species (**b**). A highly conserved NF-YA region comprises Domain A and Domain B, which are underlined. Protein sequences of the 10 tomato NF-YA members (**a**) and their counterparts from other plants were obtained from GenBank and other databases, including protein sequences for tomato (SlNF-YA3b, Solyc12g009050.1), *Arabidopsis* (AT1G72830.1), pepper (CA00g74270), eggplant (SMEL4.1_12g001770.1), tobacco (Nitab4.5_0000073g0050.1), and potato (PGSC0003DMT400000742). **c** Relative expression levels of *NF-YA3b* in different tissues of WT plants. Each statistic is displayed as a mean value ± standard error (*n* = 3). **d** Subcellular localization of an NF-YA3b-YFP fusion protein. Diagram of the construct used for subcellular localization (upper panel). TNOS, transcription termination sequence of the *Nopaline Synthase* (*NOS*) gene. Potato Ethylene Responsive Factor 3 (StERF3) tagged with an RFP served as a nuclear localization marker (StERF3-RFP) and was co-expressed transiently with NF-YA3b-YFP in tobacco leaves. Confocal microscopy was used to capture the fluorescence images. Scale bars, 36.8 μm.

In our previous work, we have shown that *NF-Y* plays a crucial role in regulating flavonoid biosynthesis in tomato [51]. To understand whether the NF-YA gene family might participate in the regulation of other important physiological and developmental processes, we performed a systematic bioinformatic analysis of the NF-YA family and investigated their possible effects on the regulation of tomato flowering. There are 10 *NF-YA* genes in the tomato genome. Protein sequence alignment of the 10 NF-YA members (SlNF-YA1a/1b/3a/3b/7a/7b/8/9/10a/10b) in tomato using MEGA10 and GeneDoc software revealed the presence of a highly conserved amino acid region among the NF-YA members (Fig. 1a). This conserved NF-YA region comprises the NF-YB/NF-YC interaction domain (Domain A) and the DNA binding domain (Domain B), separated by a short spacer of 9–10 amino acids that are also highly conserved (Fig. 1a). The presence of the conserved NF-YA region and its Domain A and Domain B structures are also found in the NF-YA counterparts from other plant species, including *Arabidopsis*, pepper, eggplant, tobacco, and potato (Fig. 1b).

In this study, we focused on *SlNF-YA3b* (Solyc12g009050), because it was found to be expressed highly in the floral primordia (Fig. 1c) and this expression pattern would imply a possible role of *SlNF-YA3b* in tomato flowering time control. The open reading frame of *SlNF-YA3b* is 762 bp long and encodes a polypeptide of 253 amino acids. The *SlNF-YA3b* gene expression profile was investigated using different tissues of WT tomato ‘Ailsa Craig’ (AC) plants. *SlNF-YA3b* was found to be expressed in all tissues tested, with the highest level of expression in the floral primordia (Fig. 1c). The expression levels of *SlNF-YA3b* were found to decrease drastically in flower buds and flowers, suggesting a role of *SlNF-YA3b* in the induction and early development of floral primordia, but not in the formation of flowers and young fruits. To investigate the subcellular localization of SlNF-YA3b, we expressed a yellow fluorescent protein (YFP)-tagged fusion protein of NF-YA3b under the CaMV35S promoter (35S:SlNF-YA3b-YFP) transiently in tobacco leaves, along with the co-expression of the nuclear localization marker of potato Ethylene Responsive Factor 3 (StERF3), which was tagged with the red fluorescent protein (RFP) under the CaMV35S promoter (35S:StERF3-RFP). As observed by confocal microscopy, the yellow fluorescent signal of SlNF-YA3b-YFP was detected in the nuclei and was found to be overlapped completely with the red fluorescent signal of the nucleus localization maker (StERF3-RFP) (Fig. 1d). The full-length coding sequence (CDS) of *NF-YA3b* was cloned into pGBKT7 vector, which was used for transformation of yeast AH109 strain to test the transcriptional activity of NF-YA3b. Transformed yeast cells with NF-YA3b-BD were grown on SD/−Trp−His medium with X-α-gal, whereas yeast cells containing the empty pGBKT7 vector did not grow well on the same medium. This result suggests that NF-YA3b has transcriptional activity to drive the expression of the *HIS3* selection marker (Supplementary Data Fig. S1). This nucleus localization pattern of SlNF-YA3b protein and the transcriptional activity of NF-YA3b are consistent with its function as a transcription factor.

### *SlNF-YA3b* negatively regulates flowering time in tomato

**Figure 2:**
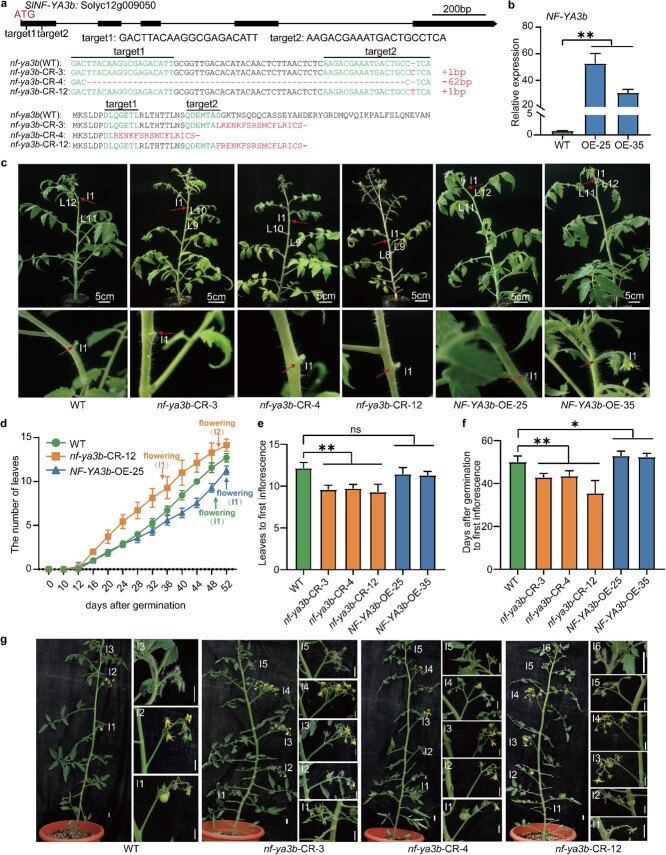
Flowering time phenotypes of *NF*-*YA3b* transgenic tomato lines. **a** Generation of *NF*-*YA3b* mutant lines (CR-3, CR-4, and CR-12) using the CRISPR/Cas9 system. Two sgRNAs (target1 and 2) used for CRISPR were designed to target exon 1 of the *NF-YA3b* gene, which contains five exons (upper panel). The nucleotide sequences of the two sgRNAs (target1 and 2) in the recipient plant (WT) of the *nf-ya3b* mutant and the knockout lines (*nf-ya3b* CR-3, 4, 12) are shown in green letters, while the CRISPR-edited sequences of the *NF-YA3b* gene are indicated by red dash symbols for base deletions and red letters for insertions (middle panel). The deduced amino acid sequences of the *NF*-*YA3b* gene in the *nf-ya3b* mutant and the knockout lines are shown in the lower panel. **b** Relative expression levels of *NF*-*YA3b* in *NF*-*YA3b*-OE lines relative to the WT. The expression level of *NF*-*YA3b* in WT was set at 1.0. **c** Eight-week-old WT, *nf-ya3b*-CR, and *NF-YA3b*-OE plants. WT, *NF-YA3b* knockout, and *NF-YA3b*-OE lines are shown. The positions of the first inflorescences (I1) and leaf (L) numbers are denoted. High-magnification images of the first inflorescence (I1) from WT, *nf-ya3b*-CR lines, and *NF-YA3b*-OE lines (lower panel). **d** Flowering time of *nf-ya3b*-CR lines (CR-12), *NF-YA3b*-OE lines (OE-25), and WT plants. The arrows indicating the first and second inflorescences’ development, respectively, represent flowering (I1) and flowering (I2). **e**, **f** Flowering time was measured relative to the number of real leaves (**e**) and relative to the number of days after seed germination (**f**) below the first inflorescences in WT, *nf-ya3b*-CR lines, and *NF-YA3b*-OE lines. Each statistic is displayed as a mean value ± standard error (*n* = 7). ns, not statistically significant; ^*^P < 0.05; ^**^P < 0.01 (*t*-test). **g** Ten-week-old WT and *nf-ya3b*-CR plants. The positions of the inflorescences are indicated with I-numbers.

To explore the function of *SlNF-YA3b* in tomato, we created both *SlNF-YA3b* knockout mutant transgenic lines and overexpression lines in the tomato AC genetic background. The *SlNF-YA3b* knockout lines were created using CRISPR/Cas9 technology. Two targets were designed on the first exon of *SlNF-YA3b*. Analysis of genomic DNA sequences in the *SlNF-YA3b* knockout lines (CR-3, CR-4, and CR-12) showed that 1 bp was inserted in *nf-ya3b*-CR-3, 62 bp were deleted in *nf-ya3b*-CR-4, and 1 bp was inserted in *nf-ya3b*-CR-12, compared with the gDNA sequence of the WT plants (Fig. 2a). The insertion or deletion of nucleotide bases in the genomic DNA of *SlNF-YA3b* in the *SlNF-YA3b* knockout lines (CR-3, -4, and -12) would result in a change in the open reading frame of *SlNF-YA3b*, leading to the loss of the function of NF-YA3b (Fig. 2a). For the overexpression lines (OE-25 and OE-35), the expression levels of *NF-YA3b* were ~52- and ~30-fold higher in OE-25 and OE-35, respectively, than that in AC plants (Fig. 2b).

Under normal growth conditions, the *NF-YA3b* knockout and overexpression lines had significant differences in flowering time from the WT plants (Fig. 2c–f and Supplementary Data Figs S2 and S3). We counted the number of real leaves under the first inflorescence and assessed the period of flowering (days) between seed germination and the first inflorescence’s development. The average number of real leaves under the first inflorescence was 9–10 in the *NF-YA3b* knockout mutant lines, compared with 12–13 in the WT plants (Fig. 2c and e). The days after seed germination for the inflorescence’s development were notably fewer in *NF-YA3b* knockout mutant lines than in the WT plants. In WT plants, it took around 48 days after seed germination for the inflorescence’s development, whereas in the *NF-YA3b* knockout lines it took just 38–44 days (Fig. 2d and f and Supplementary Data Fig. S2). Additionally, there were more inflorescences (five or six inflorescences) in the *NF-YA3b* knockout lines compared with the three inflorescences in the WT plants with the same growth time (Fig. 2g). Taken together, these results showed that *SlNF-YA3b* knockout results in early flowering, whereas *SlNF-YA3b* overexpression in tomato AC plants leads to late flowering (Fig. 2c–f and Supplementary Data Fig. S3). The number of real leaves under the first inflorescence showed no difference between the WT and *NF-YA3b*-OE lines, but the number of days after seed germination for the first inflorescence’s development were increased in the *NF-YA3b*-OE lines (Fig. 2c–f  and Supplementary Data Fig. S3). The phenotypic observation of *SlNF-YA3b* transgenic lines suggested that *SlNF-YA3b* functions as a flowering time repressor in tomato.

**Figure 3:**
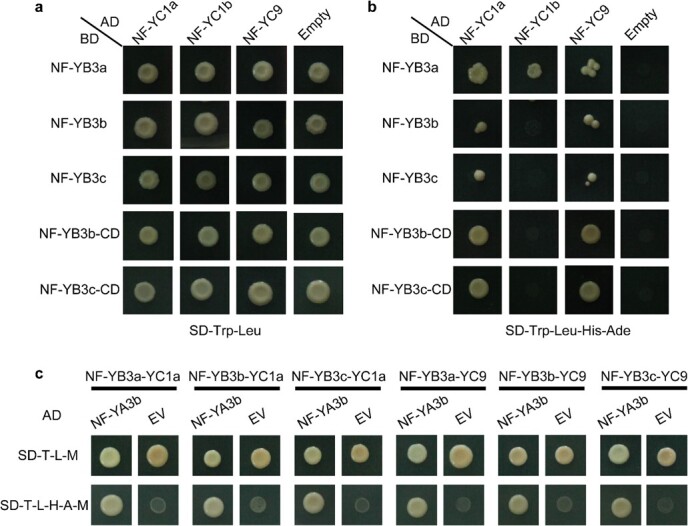
Interaction of NF-YA3b with NF-YB/NF-YC heterodimers and assembly of NF-Y complexes in yeast cells. **a**, **b** Y2H experiments for interactions among NF-YB3a/3b/3c and NF-YC1a/1b/9. NF-YB3b-CD and NF-YB3c-CD represent the truncated versions of NF-YB3b and NF-YB3c, respectively. The negative control was an empty pGADT7 vector. **c** Y3H experiments for interactions between NF-YA3b and the NF-YB3a-YC1a, NF-YB3b-YC1a, NF-YB3c-YC1a, NF-YB3a-YC9, NF-YB3b-YC9, and NF-YB3c-YC9 heterodimers. The negative control was an empty pGADT7 vector (EV).

### SlNF-YA3b was recruited by NF-YB/NF-YC heterodimers, assembling NF-Y complexes

Previous studies revealed that the motifs of NF-YB and NF-YC associate with each other in the cytoplasm, becoming a heterodimer, which subsequently translocates to the nucleus and recruits an NF-YA subunit to form the mature NF-Y heterotrimeric complex [20]. We selected three NF-YB family members (NF-YB3a/3b/3c) and three NF-YC family members (NF-YC1a/1b/9) that have reportedly been related to the regulation of flowering time in *Arabidopsis* to verify whether NF-YA3b is recruited by the interactions between the above NF-YBs and NF-YC using yeast hybrid experiments, respectively.

Analysis of toxicity tests found that the full-length NF-YB3b and NF-YB3c proteins are toxic to yeast cells (Supplementary Data Table S3). Therefore, the full-length and truncated versions of NF-YB3b and NF-YB3c were used in Y2H experiments (Fig. 3a). NF-YB3a interacted with NF-YC1a/9 and NF-YC1b. In addition, truncated versions of NF-YB3b/3c interacted with NF-YC1a/9 but not with NF-YC1b (Fig. 3a). Subsequently, we used Y3H assays to test whether the six heterodimers formed by NF-YB3a, NF-YB3b, and NF-YB3c interacting with NF-YC1a and NF-YC9 could recruit SlNF-YA3b and form the complete NF-Y complex. Yeast cells expressing SlNF-YA3b alone and the six heterodimers were cultured on yeast transformation and interaction-selection media (Fig. 3c). However, yeast cells expressing SlNF-YA3b alone and NF-YB subunits did not grow on SD/−Trp/−Leu/−His/−Ade medium (Supplementary Data Fig. S4). The above results suggested that NF-YA3b does not interact with NF-YB subunits without NF-YC subunits. However, NF-YA3b could be recruited by the NF-YB/NF-YC heterodimers to assemble into the higher-order NF-Y complexes.

### NF-Y complexes do not change *SlSFT* promoter activity

**Figure 4:**
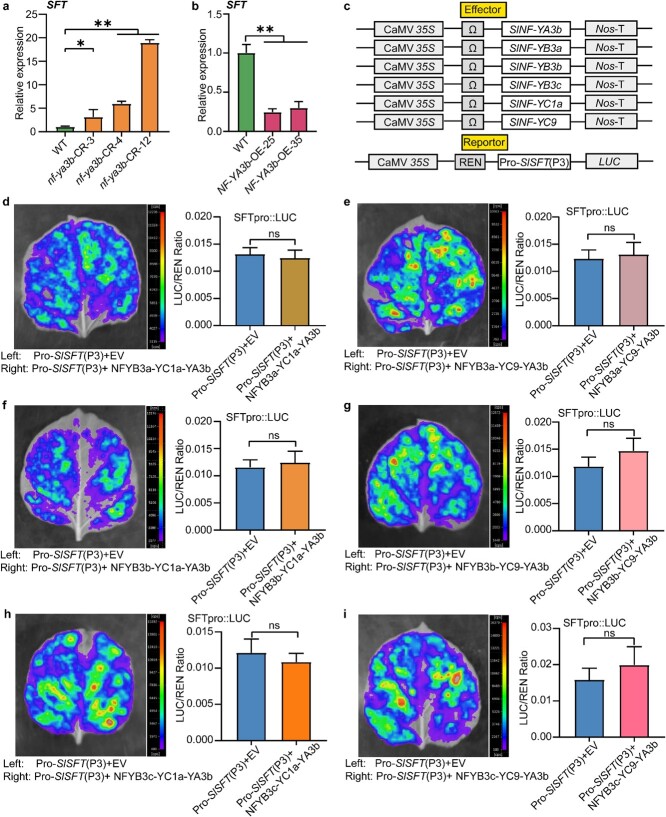
Effect of NF-Y complexes on *SlSFT* promoter activity. **a**, **b** Relative expression levels of *SFT* in *nf-ya3b*-CR lines (**a**) and *NF-YA3b*-OE lines (**b**). *SFT* gene expression in WT plants was set at 1. *n* = 3; *P < 0.05; **P < 0.01 (*t*-test). **c** Dual-luciferase reporter assays. NF-YA3b, NF-YB3a/3b/3c, and NF-YC1a/9 were expressed from pGreenII 62-SK with the CaMV 35S promoter and used as the effector. Pro-*SlSFT* (P3):LUC served as the reporter and was expressed from pGreenII 0800-LUC. Pro-*SlSFT* (P3) was a fragment of the *SFT* promoter containing boxes 1–7, which is 4150 bp in length upstream of the translation start site (TSS). **d**–**i** Representative images of luciferase activity (left) and ratios of firefly luciferase (LUC)/*Renilla* luciferase (REN) activities (right). In the control, the empty vector (EV) was used as the effector. Co-expression of the reporter Pro-*SlSFT* (P3):LUC with different effector vectors (NFYB3a-YC1a-YA3b, NFYB3a-YC9-YA3b, NFYB3b-YC1a-YA3b, NFYB3b-YC9-YA3b, NFYB3c-YC1a-YA3b, and NFYB3c-YC9-YA3b) was examined in *N. benthamiana* leaves. Values are expressed as mean ± standard error (*n* = 8); ns, not statistically significant (*t*-test).

SFT is the ortholog of *Arabidopsis* FT and is considered to be the putative florigen in tomato [21, 32]. There are ~20 true leaves below the first inflorescence in the *sft* mutant compared with only 10–12 leaves in the control plants [36]. Thus, *sft* is considered a late-flowering mutant of tomato [36]. Overexpression of *SFT* led to early flowering, developing the first inflorescence after three to five real leaves [31]. In this work, *nf-ya3b* mutant lines and *SFT* overexpression lines were found to have similar phenotypes in flowering time (Fig. 2). Therefore, we speculated that *NF-YA3b* may negatively regulate the expression of *SFT*. To test this idea, we detected the expression levels of the *SFT* gene in the *nf-ya3b* mutant lines. *SFT* expression was significantly up-regulated in the *nf-ya3b* mutant lines (Fig. 4a) and down-regulated in the *NF-YA3b*-OE lines (Fig. 4b), suggesting that *SlNF-YA3b* controls tomato flowering time by inhibiting *SFT* expression.

Previous studies have shown that the NF-Y complex functions by recognizing and binding the key *cis*-regulatory elements on the target gene promoter by the NF-YA subunit [24]. Our results of Y3H experiments showed that SlNF-YA3b could be recruited by the NF-YB/NF-YC heterodimer to form the NF-Y complex (Fig. 3). To verify whether the NF-Y complex could regulate *SFT* expression, we performed dual-luciferase reporter experiments. Co-expression of *SFT*pro:LUC with any NF-Y complex had no significant effect on LUC activity compared with LUC activity in transgenic plants expressing the empty vector (Fig. 4c–i), suggesting that NF-Y complexes do not change *SFT* promoter activity.

### *SlNF-YA3b* binds to the CCAAT element of the *SFT* promoter and represses its expression

**Figure 5:**
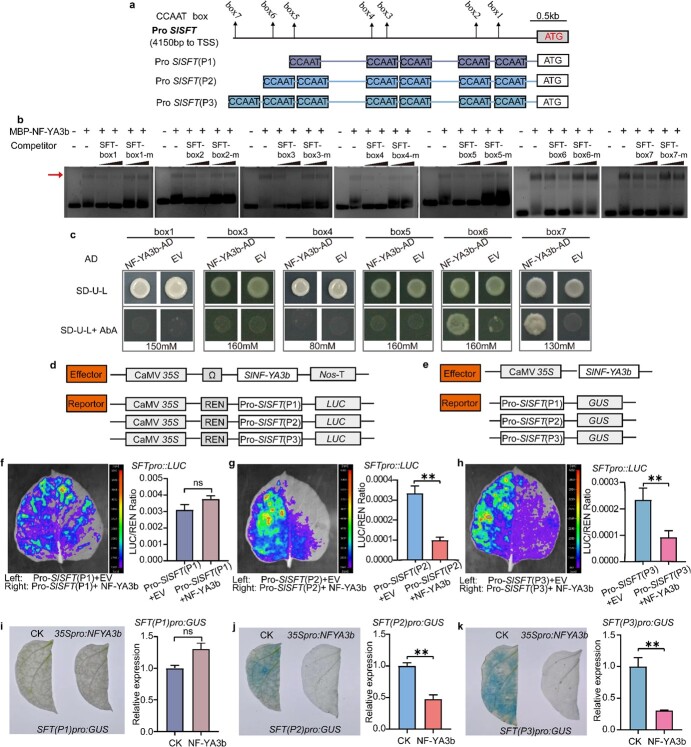
Specific binding of SlNF-YA3b to the CCAAT *cis*-elements of the *SFT* promoter. **a** Schematic diagram of the 4150-bp *SFT* promoter region. Seven CCAAT (boxes 1–7) *cis-*elements were identified in the 4150-bp fragment of the *SFT* promoter. The translation start codon (ATG) is indicated. TSS, translation start site. Pro *SlSFT* (P1), Pro *SlSFT* (P2), and Pro *SlSFT* (P3) constitute a genomic fragment of the *SFT* promoter containing boxes 1–5, boxes 1–6, and boxes 1–7, respectively. They are 3589, 3627, and 4150 bp in length upstream of the TSS. **b** EMSAs for binding of NF-YA3b to the CCAAT *cis*-elements in boxes 1/2/3/4/5/6/7 of the *SFT* promoter. NF-YA3b protein was incubated with FAM-labeled box 1/2/3/4/5/6/7 probes, and the mobilities of the protein–oligonucleotide probe complexes on non-denaturing gels are indicated with red arrows. The specific unlabeled competitors (SFT-box1/2/3/4/5/6/7) or unlabeled mutant oligonucleotide competitors (SFT-box1/2/3/4/5/6/7-m) were added to the incubation mixtures of lanes 3, 4, 5, and 6, by a 10- and 30-fold molar excess. −, absence; +, presence. **c** Y1H experiments for the binding of NF-YA3b to the CCAAT *cis*-element at different positions of the *SFT* promoter. Seven constructs containing individual CCAAT *cis-*elements were used in the assays. The negative control was an empty pGADT7 vector (EV). **d** Dual-luciferase reporter assays. *NF-YA3b* was expressed from pGreenII 62-SK under the CaMV 35S promoter and served as an effector. Pro *SlSFT* (P1), Pro *SlSFT* (P2), and Pro *SlSFT* (P3) were cloned into the pGreenII 0800-LUC vector and used to drive the expression of the LUC reporter. **e** GUS gene expression assays. The full-length CDS of *NF-YA3b* was cloned into pHellsgate8 for expression of NF-YA3b protein as the effector. Three *SFT* promoter fragments of 3589 bp (*SFT*-P1), 3627 bp (*SFT*-P2), and 4150 bp (*SFT*-P3) upstream of the translation start codon (ATG) were cloned into pMV2-GUS vector to drive *GUS* gene expression. **f**–**h** Representative images of luciferase activity (left) and ratios of LUC/REN activities (right) in *N. benthamiana* leaves. Note that SlNF-YA3b suppresses its transcriptional activity on *SlSFT* (P2) and *SlSFT* (P3) but does not change its transcriptional activity on *SlSFT* (P1). The values displayed are mean ± standard error (*n* = 8). ns, not statistically significant; ^**^P < 0.01 (*t*-test). **i**–**k** Representative images of GUS activity staining (left) and relative GUS gene expression levels (right) in *N. benthamiana* leaves. In the control (CK), the empty pHellsgate8 vector was used to replace the effector for co-expression with *SFT(P1)pro:GUS* (**i**), *SFT(P2)pro:GUS* (**j**), and *SFT(P3)pro:GUS* (**k**) in *N. benthamiana* leaves. The *GUS* gene expression level in the control (CK) was set as 1.0. Data are mean ± standard error (*n* = 3). ns, not statistically significant, ^**^P < 0.01 (*t*-test).

Previous findings have shown that the NF-YA subunit of NF-Y complexes is the protein component that specifically binds to the CCAAT box of the promoters of target genes [38]. Seven CCAAT *cis*-elements (boxes 1–7) were identified in the 4150-bp genomic DNA fragment upstream of the *SFT* promoter (Fig. 5a). To find out whether NF-YA3b could bind to the CCAAT *cis*-element of the *SFT* promoter *in vitro*, we performed EMSA assays. In these assays, NF-YA3b protein was expressed in and purified from *Escherichia coli*. Seven oligonucleotides of 39 bp each, containing the core CCAAT box 1, 2, 3, 4, 5, 6, and 7 sequences from the *SFT* promoter, respectively, were labeled with the fluorescent dye 6-carboxyfluorescein (FAM), which absorbs 495-nm light and emits a 517-nm signal. Unlabeled oligonucleotides were used as the competitive binding targets. To assess the specificity of binding, mutant oligonucleotides where the CCAAT *cis*-element was changed to CCCCC or AAAAA were used as unlabeled mutant oligonucleotide competitors. When NF-YA3b protein was incubated with FAM-labeled probes corresponding to sequences of boxes 1, 2, 3, 4, 5, 6, and 7, DNA–protein complexes were detected with retarded mobility on EMSA, suggesting that NF-YA3b binds to the seven CCAAT *cis*-elements (boxes 1–7) of the *SFT* promoter (Fig. 5b). When an excessive and increasing amount of unlabeled competitive probes was added to the assays, the signals of the DNA–protein complexes decreased or even disappeared, whereas when the same amount of mutant oligonucleotides (SFT boxes 1/2/3/4/5/6/7-m) was added, the signals of the DNA–protein complexes did not change (Fig. 5b), suggesting that NF-YA3b recognizes and binds to the specific DNA sequence of CCAAT in the 4150-bp genomic DNA fragment upstream of the *SFT* promoter. In conclusion, these results indicate that NF-YA3b directly binds to the seven CCAAT *cis*-elements in the 4150-bp genomic DNA fragment upstream of the *SFT* promoter *in vitro*.

We also carried out Y1H assays to clarify whether *NF-YA3b* could bind to the CCAAT *cis*-element of the *SFT* promoter in yeast cells. When NF-YA3b and individual CCAAT *cis*-elements (boxes 1–7) were co-expressed in yeast cells, it was found that the *SFT* promoters containing box 6 and box 7 conferred antibiotic resistance in the presence of 160 and 130 mM aureobasidin A (AbA), respectively (Fig. 5c). In contrast, when the *SFT* promoters containing boxes 1, 3, 4, and 5, or no CCAAT *cis*-element (negative control) were used, the yeast cells were unable to grow in the presence of the antibiotic AbA (Fig. 5c), suggesting that NF-YA3b could not bind to the *SFT* promoter containing boxes 1, 3, 4, and 5, thus failing to activate the expression of the antibiotic resistance gene. The *SFT* promoter containing box 2 was ‘toxic’ to yeast cells for unknown reasons and yeast cells containing this construct were unable to grow on transformation-selection medium SD/−Ura−Leu without AbA (data not shown). Taken together, these Y1H assay results showed that NF-YA3b specifically binds to CCAAT boxes 6 and 7 of the *SFT* promoter.

To further determine whether NF-YA3b could bind to the CCAAT *cis*-elements of the *SFT* promoter to regulate its gene expression *in planta*, we conducted dual-luciferase reporter and GUS expression assays using NF-YA3b as the effector. *NF-YA3b* was cloned into the pGreenII 62-SK effector vector. Three *SFT* promoter fragments of 3589 bp (*SFT*-P1), 3627 bp (*SFT*-P2), and 4150 bp (*SFT*-P3) upstream of the translation start codon (ATG) were cloned into the pGreenII 0800 LUC reporter vector (Fig. 5a and d). The dual-luciferase reporter results showed that NF-YA3b did not change the expression levels of the *Luc* reporter gene under the *SFT* promoter *SFT*-P1, which contained boxes 1–5 (Fig. 5f). However, NF-YA3b was found to significantly suppress *Luc* reporter gene expression under either the *SFT* promoter *SFT*-P2 or *SFT*-P3, which contained boxes 1–6 and 1–7, respectively (Fig. 5g and h). Similarly, the results of the GUS activity assay were consistent with those of the dual-luciferase reporter assays (Fig. 5e). When *SFT(P1)pro:GUS* and *35Spro:NFYA3b* were co-expressed, the GUS gene expression levels were not significantly different from the control, which co-expressed *SFT(P1)pro:GUS* with the empty pHellsgate8 (CK) vector (Fig. 5i). However, when *SFT(P2)pro:GUS* or *SFT(P3)pro:GUS* was co-expressed with *35Spro:NFYA3b* (Fig. 5j and k), significantly decreased GUS gene expression levels were observed. Taken together, these findings indicate that NF-YA3b binds to CCAAT boxes 6 and 7 of the *SFT* promoter and acts as a transcriptional suppressor of *SFT* gene expression *in planta.*

## Discussion

NF-Ys have been demonstrated to regulate a wide range of biological processes in plants [7, 22, 23, 25, 40, 41, 43]. One of these NF-Y-regulated processes is flowering time control in flowering plants [24, 41, 47]. NF-YA, a subunit of the NF-Y tri-protein complex, plays a pivotal role in the regulation of flowering time in plants [5, 6, 24, 41]. In *Arabidopsis*, overexpression of any of the *NF-YA1*/*3*/*4*/*5*/*7*/8/*9*/*10* genes delays flowering [26, 37, 54, 62]. *AtNF-YA2* and *AtNF-YA6* are positive regulators of flowering by activating *FT* gene expression [47]. Extensive functional redundancy of the *NF-YA* family and the embryo lethality of multiple mutants dramatically limit the possibility of *NF-YA* mutants in studies on flowering time control [13, 37, 46]. Therefore, for single mutations of these *NF-YA* members, there have been no reports of a significantly early flowering phenotype. Here, we identified a tomato *nf-ya3b* mutant with an early flowering phenotype. Knockout of the *NF-YA3b* gene by CRISPR-Cas 9 technology was found to promote flowering in tomato. In contrast, overexpression of *NF-YA3b* led to delays in flowering in transgenic tomato plants. These results suggested that *NF-YA3b* is a negative flowering regulator in tomato, which agrees with the findings of previous studies in *Arabidopsis* [26, 37, 54]. Notably, our findings fill the gap of a single *nf-ya3b* mutant causing early flowering in tomato.

The NF-Y transcriptional activator is a heterotrimeric complex formed by three different subunits: NF-YA, NF-YB, and NF-YC [3, 5, 22]. NF-YB and NF-YC form heterodimers in the cytoplasm and subsequently translocate to the nucleus, recruiting NF-YA to form heterodimers [20]. The NF-YA/YB/YC complex regulates the expression of target genes through a mechanism by which the NF-YA subunit of the heterotrimeric complex recognizes and binds to the CCAAT *cis*-element of gene promoters [24]. In this study, the results from Y2H and Y3H assays showed that NF-YA3b was recruited by the NF-YB/NF-YC heterodimers, assembling into higher-order NF-Y heterotrimeric complexes (Fig. 3). However, in dual-luciferase experiments we found that these NF-Y complexes did not affect the expression of the luciferase (*Luc*) reporter gene under the *SFT* promoter (Fig. 4c–i). We speculate that these NF-Y complexes might regulate other target genes? involved in processes that are unrelated to flowering time control. Interestingly, the individual NF-YA3b could lead to more than 2-fold decreases in the expression of the *SFT*-*Luc* reporter gene (Fig. 5g–h). Consistently, numerous single NF-Y subunits have been shown to bind to the CCAAT *cis*-element in the absence of the other two NF-Y subunits [2, 7, 45, 56]. By binding to the CCAAT *cis*-element of *AtXTH21*, *AtHAP5A* (an *NF-YC* gene) affects freezing stress resistance in *Arabidopsis* [45]. The aleurone-specific *NF-YB1*, which is more abundant on the dorsal side, is critical in regulating rice grain fullness by stimulating the expression of the *Sucrose Transporter* (*SUT*) genes *SUT1*/*3*/*4* [2]. In rice, *NF-YC12* directly binds to the *FLOURY ENDOSPERM6* (*FLO6*) and *Glutamine Synthetase1* (*OsGS1;3*) promoters and regulates endosperm development [56]. In this study, NF-YA3b was demonstrated to bind to the *SFT* promoter without NF-YB and NF-YC subunits (Fig. 5).

NF-Y transcription factors are heterotrimeric complexes that can change their activities depending on the compositions of the three subunits. Therefore, there is wide variability in the biological activity of NF-Y in plants, and there are various possibilities for opposing regulations at a single DNA binding site [23, 26]. Numerous studies have demonstrated that positive regulators of *FT* expression are known as NF-Ys in *Arabidopsis*. *AtNF-YA2*, *AtNF-YA6*, *AtNF-YB2*, *AtNF-YB3*, *AtNF-YC1*, and *AtNF-YC2* have been shown to promote flowering by inducing the expression of *FT* [16, 22, 47]. Additionally, there have been reports presenting strong evidence for the negative effect of NF-Ys on gene expression [39, 54, 57]. Overexpression of *AtNF-YA1*, *AtNF-YA4*, and *AtNF-YB1* has been shown to down-regulate *FT* expression, resulting in delayed flowering [39, 54]. *AtNF-YA4*/*5*/*7*/*9* have negative effects on the expression of several ABA-responsive genes by blocking the interaction between NF-YB/NF-YC dimers and bZIP family members [57]. *AtNF-YA8* suppresses the expression of a subset of age-dependent genes, negatively regulating flowering time [62]. *SlNF-YA10* negatively regulates ascorbate accumulation by binding to the *SlGGP1* promoter and inhibiting its expression [9]. In this study, we provide genetic and biochemical evidence that NF-YA3b binds to the CCAAT *cis-*element of the *SFT* promoter and suppresses its expression (Fig. 5). *SFT* is the florigen gene in tomato, which positively regulates flowering time. The *sft* mutant is known to produce flowers later than WT tomato plants [32, 36]. Consistent with this notion of *SFT* in flowering control, there has been a report showing that overexpression of *SFT* results in earlier flowering after three to five true leaves in tomato [30]. Consistent with the conclusion of these reports, our data showed that the *SlNF-YA3b* knockout lines exhibited early flowering (Fig. 2) and the expression level of the *SFT* gene was significantly up-regulated (Fig. 4a). Taken together, these results imply that *NF-YA3b* acts as a transcriptional repressor of *SFT*, negatively controlling flowering time in tomato. Remarkably, to our knowledge, this is the first direct demonstration that the individual NF-Y subunit directly binds to the *SFT* promoter and functions as a repressor of *SFT* transcription.

**Figure 6:**
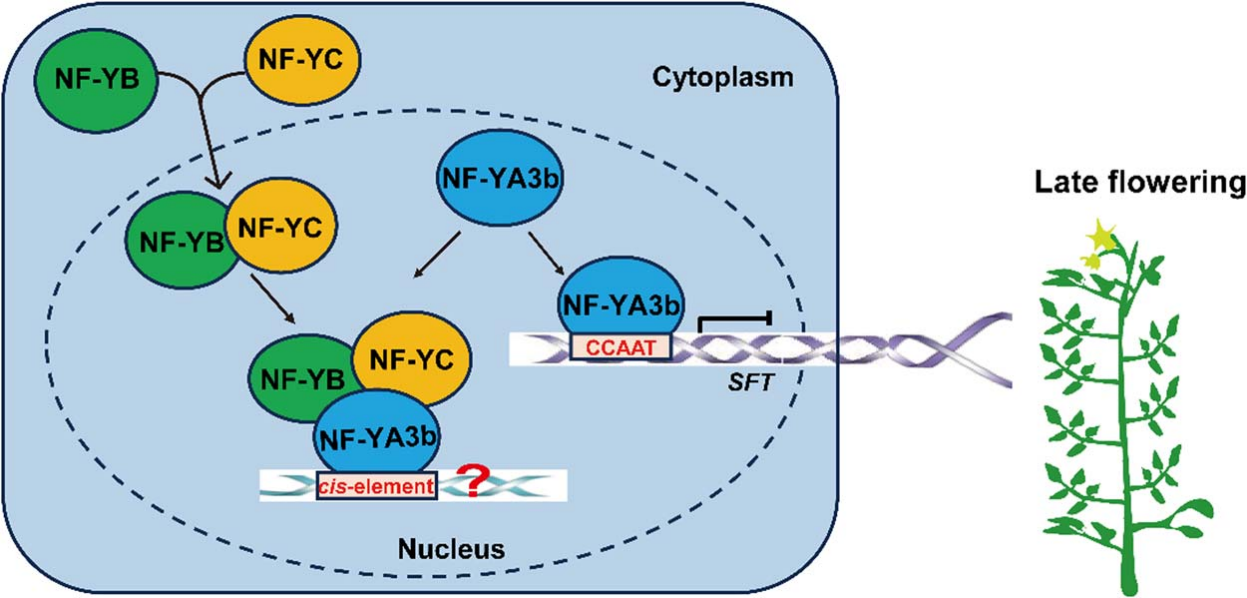
A model for the regulation of tomato flowering by *NF-YA3b.* In this model, NF-YB and NF-YC form heterodimers in the cytoplasm and move to the nucleus, where they recruit NF-YA3b to form heterotrimer protein complexes. The NF-YB/YC/YA3b complex may bind to the promoters of other target genes and be involved in the regulation of other pathways. On the other hand, NF-YA3b may bind directly to the CCAAT *cis*-element of the *SFT* promoter and suppress its gene expression, leading to late flowering in tomato.

A model is proposed based on our findings that *NF-YA3b* controls flowering time by repressing *SFT* expression in tomato (Fig. 6). In this model, NF-YB and NF-YC form heterodimers in the cytoplasm and subsequently move to the nucleus, recruiting NF-YA3b to form heterodimers. NF-YA3b directly binds to the CCAAT *cis-*element of the *SFT* promoter and suppresses its gene expression, leading to late flowering in tomato. We speculate that the NF-YB/YC/YA3b heterotrimeric protein complex may bind to the promoters of other target genes, participating in the regulation of gene expression in other physiological and developmental pathways. There also exists a possibility that loss of function of *NF-YA3b* may promote flowering through a different mechanism by which the up-regulation of *SFT* expression is caused via regulation by transcription activation mediated by CO or other transcription factors. In summary, the results from this work have advanced our understanding of the regulatory mechanism involved in tomato flowering, which could be applied to crop improvement and germplasm innovation.

## Materials and methods

### Plant materials and growth conditions

In this study, the tomato variety ‘Ailsa Craig’ (AC) served as the wild-type (WT) and was used for background plants in stable transformation of tomato. For *Agrobacterium*-mediated transient transformation experiments, *Nicotiana benthamiana* was utilized. All plants were grown in the greenhouse under conditions that included 23°C ambient temperature, 60–75% relative humidity, and a photoperiod of 16 h of natural daylight followed by 8 h of darkness.

### Subcellular localization

The total cDNA of AC plants was used as a template for amplification of the CDS of *SlNF-YA3b* excluding the stop codon. The amplified *SlNF-YA3b* CDS was fused with the coding sequence for YFP to generate 35S:SlNFYA3b-YFP for expression of the NF-YA3b-YFP fusion protein for subcellular localization experiments. Plasmid 35S:StERF3-RFP, which expressed the fusion protein of potato Ethylene Responsive Factor 3 (StERF3)–RFP, served as a nuclear localization marker. 35S:SlNFYA3b-YFP and 35S:StERF3-RFP were expressed together in tobacco leaves. Two days after infiltration with *Agrobacterium* strains carrying the appropriate plasmids, leaves expressing fluorescent fusion proteins were obtained using a confocal laser scanning microscope (Leica TCS-SPE).

### RNA extraction and gene expression profiling

Total RNAs were extracted from various tomato tissues using the TRIzol^®^ 117 reagent (Invitrogen). A reverse transcription kit (Vazyme, Nanjing, China) was utilized to generate single-stranded cDNA using 2 μg of total RNA. Gene expression assays by qPCR analysis were performed using the ChamQ SYBR Color qPCR Master Kit (Vazyme, Nanjing, China). All data had three biological replicates and were analyzed. The internal control was the expression of the *Actin* gene (Solyc11g005330). Supplementary Data Table S1 lists the primer sequences used in real-time PCR.

### Vector construction and tomato genetic transformation

For the generation of the *SlNF-YA3b* overexpression construct, the CDS of *SlNF-YA3b* was amplified from AC and connected to the pHellagate 8 vector driven by the CaMV35S promoter. For the generation of the *SlNF-YA3b* CRISPR/Cas9 construct, two sgRNAs in the first exon of *SlNF-YA3b* were designed by CRISPR-direct web (http://crispr.dbcls.jp) and linked to pTX041 vector following a method described previously [59]. *SlNF-YA3b* CRISPR/Cas9 and overexpression constructs were introduced into the WT tomato AC plants using an *Agrobacterium*-mediated stable transformation system.

### Yeast two-hybrid assays

The Matchmaker GAL4-based Yeast Two-Hybrid System (Clontech, CA, USA) was used for verifying protein–protein interactions. The full-length CDSs of *NF-YB3a*/*3b*/*3c* and truncated CDS of *NF-YB3b*/*3c* were amplified and linked to pGBKT7 vectors. The full-length CDSs of *NF-YC1a*/*1b*/*9* and *NF-YA3b* were amplified and then linked into pGADT7 vectors. *Saccharomyces cerevisiae* strain AH109 was co-transformed with the pairs of plasmids. Yeast cells were cultured on transformation-selection (SD/−Leu−Trp) and interaction-selection (SD/−Leu-Trp−Ade−His) medium to screen for protein–protein interactions. Specific primers (Supplementary Data Table S1) were used to construct the plasmids.

For transcriptional activation assays in yeast, the full-length CDS of *NF-YA3b* was cloned into pGBKT7 vector, and the resulting construct was used for transformation of yeast AH109 strain. The empty vector pGBKT7 and the combination of pGBKT7-53 + pGADT7-RecT vectors were used as the negative and positive controls, respectively. Transformed yeast cells were grown on SD/−Trp, SD/−Trp−His, and SD/−Trp−His with X-α-gal media.

### Yeast three-hybrid assays

Y3H assays were conducted using the pBridge system (Clontech) to test the interactions between NF-YA3b and NF-YBs-YCs (including NF-YB3a-YC1a, NF-YB3b-YC1a, NF-YB3c-YC1a, NF-YB3a-YC9, NF-YB3b-YC9, and NF-YB3c-YC9). There are two multiple cloning sites in the pBridge system. The full-length CDSs of NF-YCs and NF-YBs were separately linked to the pBridge vectors, while the NF-YA3b CDS was linked to pGADT7 vector. The pairs of plasmids were co-transformed into yeast strain AH109, and the transformed yeast cells were cultured on SD/−Leu−Trp−Met before selecting them for protein interactions on SD/−Leu−Trp−Ade−His−Met media.

### Yeast one-hybrid assays

For Y1H assays, the Matchmaker Gold One-Hybrid Library Construction & Screening Kit (Clontech) was utilized. There are seven CCAAT *cis*-elements in the 4.3-kb *SFT* promoter. The promoter fragments containing each CCAAT *cis*-element with surrounding sequences were amplified and inserted into pAbAi. The NF-YA3b CDS was linked to pGADT7. Yeast strain Y1H Gold was co-transformed with the pairs of plasmids. Yeast cells were cultured on SD/−Ura−Leu and selected for promoter activities on selection medium (SD/−Ura−Leu) containing different concentrations of AbA.

### Protein expression and electrophoretic mobility shift assay

The CDS of *NF-YA3b* was amplified and then ligated into pET15d-MBP, expressing the recombinant protein with a maltose-binding protein (MBP) tag and 6-His tags. The plasmid was subsequently transformed into *E. coli* DE3 cells. The proteins were purified following the method described previously [27].

Two *SFT* promoter fragments each containing a CCAAT *cis*-element (box 6 and box 7) were used as oligonucleotide probes. The probes were labeled with FAM and synthesized by Tianyi (Wuhan, China). Mutant probes contained CCCCC to replace CCAAT in box 6 or GGGGG to replace ATTGG in box 7 and were used as competitor probes in EMSA, following a previously described method [34].

### Dual-luciferase reporter assays for transcription activities

The CDS of *NF-YA3b* was cloned into the pGreenII 62-SK effector vector under the CaMV35S promoter. Three genomic fragments of 3589 bp (containing boxes 1–5 *cis*-elements), 3627 bp (containing boxes 1–6 *cis*-elements), and 4150 bp (containing boxes 1–7 *cis*-elements) upstream of the start codon from the *SFT* promoter were inserted into pGreenII 0800-LUC reporter vector. The pairs of constructs were transformed into *Agrobacterium tumefaciens* strain GV2260 cells with the helper plasmid pSoup19. Co-expression of the effector and reporter vectors in tobacco leaves was performed via *Agrobacterium*-mediated transformation following a method described previously [51]. Tecan’s Infinite 200 Pro microplate reader and the Dual-Luciferase Reporter Assay System (Promega, USA) were used to measure the activities of firefly luciferase (LUC) and *Renilla* luciferase (REN). Luciferin (1 mM, Gold Biotech, Olivette, MO, USA) was sprayed onto leaves of *N. benthamiana* to detect firefly LUC activity using the NightSHADE LB 985 system (Berthold, Bad Wildbad, Germany).

### GUS activity assay

The full-length CDS of *NF-YA3b* was cloned into pHellsgate8 for expression of the effector. Three *SFT* promoter fragments of 3589 bp (SFT-P1), 3627 bp (SFT-P2), and 4150 bp (SFT-P3) upstream of the start codon were cloned into pMV2-GUS to drive the expression of the *GUS* reporter. The reporter and effector constructs were co-expressed in *N. benthamiana* leaves in the *Agrobacterium*-mediated transient expression system. Three days after *Agrobacterium* infiltration, the leaves were submerged in GUS staining buffer with 2 mM X-glucuronide at 37°C for 24 h, followed by washing with 95% ethanol. Relative expression levels of the *GUS* gene were measured by qRT–PCR.

### Statistical analysis

GraphPad Prism 8.0 and Excel were both utilized for statistical analysis. Student’s *t*-test was employed to compare groups in pairs. Two categories have been defined for statistically significant differences: *P* < 0.05 and *P* < 0.01.

## Supplementary Material

Web_Material_uhae088

## Data Availability

The data on which this article is based can be found in this article and its online supplement. The sequences of the genes presented in this study can be accessed in the Sol Genomics Network (http://solgenomics.net/) under the following accession numbers: *SlNF-YA3b*, Solyc12g009050; *SlNF-YA1a*, Solyc01g008490; *SlNF-YA1b*, Solyc11g065700; *SlNF-YA3a*, Solyc03g121940; *SlNF-YA7a*, Solyc02g069860; *SlNF-YA7b*, Solyc10g079150; *SlNF-YA8*, Solyc08g062210; *SlNF-YA9*, Solyc01g087240; *SlNF-YA10a*, Solyc01g006930; *SlNF-YA10b*, Solyc10g081840; *SlNF-YB3a*, solyc04g054150; *SlNF-YB3b*, solyc07g065500; *SlNF-YB3c*, solyc12g006120; *SlNF-YC1a*, solyc03g110860; *SlNF-YC1b*, solyc03g111450; *SlNF-YC1d*, solyc06g072040; *SlNF-YC9*, solyc01g079870; *SFT*, Solyc03g063100; *Actin*, Solyc11g005330. The protein sequences used for multiple sequence alignment analysis are listed in Supplementary Data Table S2.
