# Supplementary material for: Nuclear factor Y-A3b binds to the SINGLE FLOWER TRUSS promoter and regulates flowering time in tomato
Source: Hortic Res. 2024 Apr 2;11(5):uhae088. doi: 10.1093/hr/uhae088 (PMC11116822; doi:10.1093/hr/uhae088)
Supplement: Web_Material_uhae088 [file web_material_uhae088.zip › Revised Fig. S.docx]

**Supplemental Figures and figure legends**


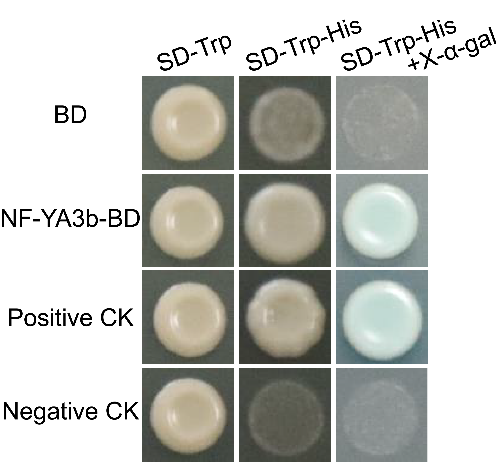


**Fig. S1 Transcription activity of NF-YA3b in yeast.**

Transcription activation assays in yeast cells. *NF-YA3b* was cloned in pGBKT7 vector for expression of the fusion protein with the DNA-binding domain (BD) of the yeast GAL4 transcription factor. If NF-YA3b has transcription activation activity, yeast AH109 cells containing the NF-YA3b expression vector would grow on SD/-Trp-His selection medium and express *lacZ* reporter gene. BD, yeast AH109 containing pGBKT7 empty vector. Positive CK, positive control of yeast AH109 containing pGBKT7-53 + pGADT7-RecT constructs (Clontech, CA, USA). Yeast AH109 cells containing the appropriate plasmid was cultured on SD/-Trp medium and selected for growth on SD/-Trp-His agar-medium or for β-galactosidase activity on SD/-Trp-His with X-α-gal media.


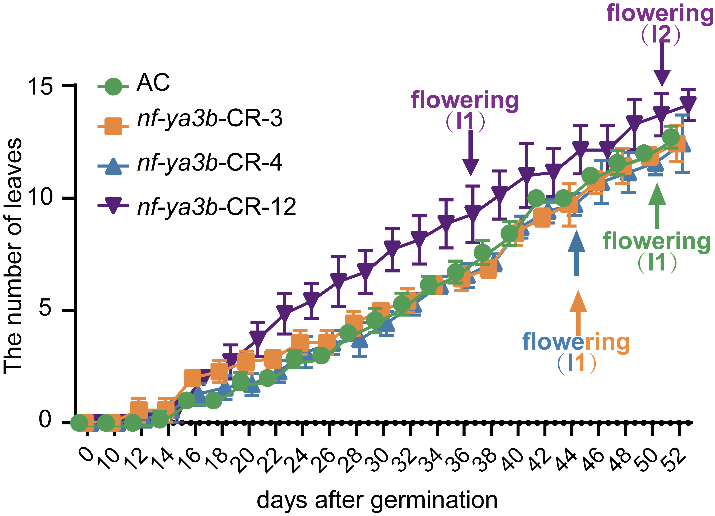


**Fig. S2 Flowering time of *nf-ya3b*-CR lines and WT tomato (AC) plants.**

The numbers of leaves in *nf-ya3b*-CR lines (CR-3, -4, and -12) and WT tomato (AC) plants were recorded continuously for days after germination.


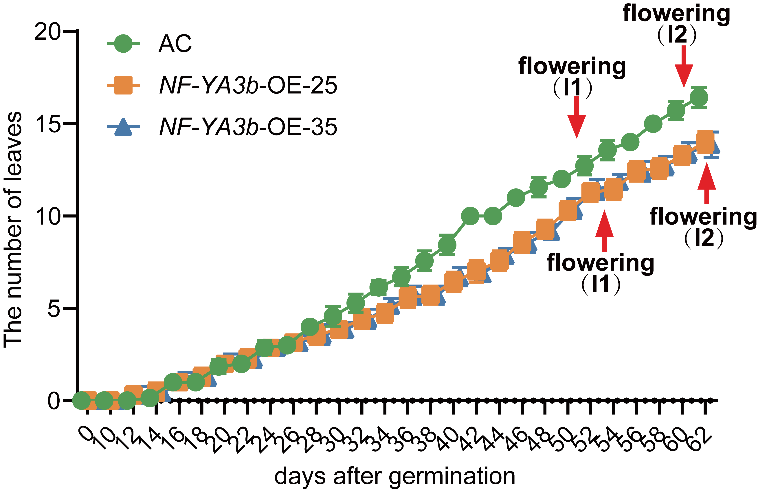


**Fig. S3 Flowering time of *NF-YA3b*-OE lines and WT tomato (AC) plants.**

The numbers of leaves in *NF-YA3b*-OE lines (OE-25 and -35) and WT tomato (AC) plants were recorded continuously for days after germination.


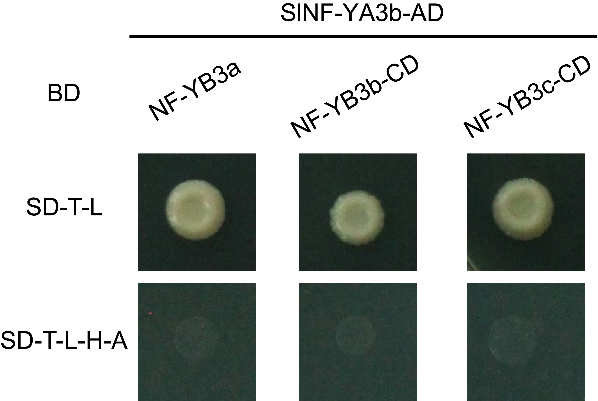


**Fig. S4** **Lack of interaction between NF-YA3b and NF-YB subunits in yeast.**

Y2H experiments for interactions between NF-YA3b and NF-YB3a, NF-YB3b, and NF-YB3c, respectively. NF-YB3b-CD and NF-YB3c-CD represent the truncated versions of NF-YB3b and NF-YB3c, respectively.
